# Radiolabeled nanomaterials for biomedical applications: radiopharmacy in the era of nanotechnology

**DOI:** 10.1186/s41181-022-00161-4

**Published:** 2022-04-25

**Authors:** Martha Sahylí Ortega Pijeira, Herlys Viltres, Jan Kozempel, Michal Sakmár, Martin Vlk, Derya İlem-Özdemir, Meliha Ekinci, Seshasai Srinivasan, Amin Reza Rajabzadeh, Eduardo Ricci-Junior, Luciana Magalhães Rebelo Alencar, Mohammed Al Qahtani, Ralph Santos-Oliveira

**Affiliations:** 1 Laboratory of Nanoradiopharmaceuticals and Synthesis of Novel Radiopharmaceuticals, Nuclear Engineering Institute, Brazilian Nuclear Energy Commission, Rua Helio de Almeida, 75, Ilha Do Fundão, Rio de Janeiro, RJ 21941906 Brazil; 2 School of Engineering Practice and Technology, McMaster University, 1280 Main Street West, Hamilton, ON L8S 4L8 Canada; 3 Department of Nuclear Chemistry, Faculty of Nuclear Sciences and Physical Engineering, Czech Technical University in Prague, Břehová 7, 11519 Prague 1, Czech Republic; 4 Department of Radiopharmacy, Faculty of Pharmacy, Ege University, 35040 Bornova, Izmir, Turkey; 5 School of Pharmacy, Federal University of Rio de Janeiro, Rio de Janeiro, 21940000 Brazil; 6 Laboratory of Biophysics and Nanosystems, Department of Physics, Federal University of Maranhão, Campus Bacanga, São Luís, Maranhão 65080-805 Brazil; 7 Cyclotron and Radiopharmaceuticals Department, King Faisal Specialist Hospital & Research Centre, Riyadh, 11211 Saudi Arabia; 8 Laboratory of Radiopharmacy and Nanoradiopharmaceuticals, State University of Rio de Janeiro, Rio de Janeiro, 23070200 Brazil

**Keywords:** Radiolabeled nanoparticles, Technetium-99m, Copper-64, Lutetium-177, Radium-223, Molecular imaging, Radionuclide therapy, Theranostics, Toxicity, Radiopharmacy

## Abstract

**Background:**

Recent advances in nanotechnology have offered new hope for cancer detection, prevention, and treatment. Nanomedicine, a term for the application of nanotechnology in medical and health fields, uses nanoparticles for several applications such as imaging, diagnostic, targeted cancer therapy, drug and gene delivery, tissue engineering, and theranostics.

**Results:**

Here, we overview the current state-of-the-art of radiolabeled nanoparticles for molecular imaging and radionuclide therapy. Nanostructured radiopharmaceuticals of technetium-99m, copper-64, lutetium-177, and radium-223 are discussed within the scope of this review article.

**Conclusion:**

Nanoradiopharmaceuticals may lead to better development of theranostics inspired by ingenious delivery and imaging systems. Cancer nano-theranostics have the potential to lead the way to more specific and individualized cancer treatment.

**Graphical abstract:**

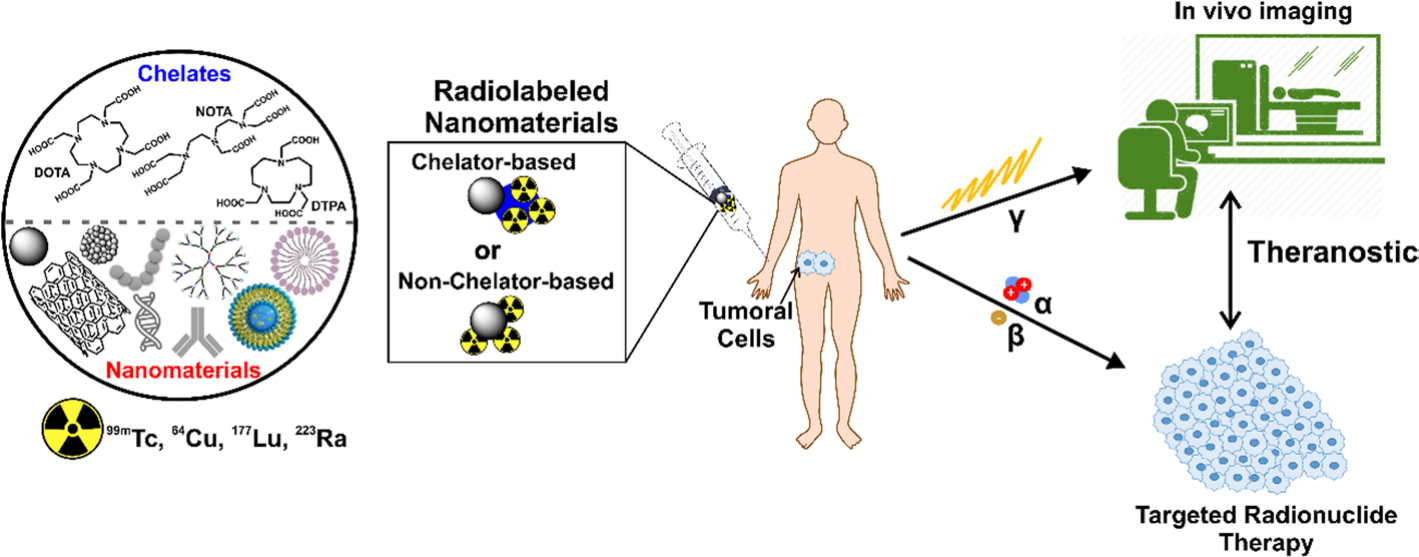

## Background

Since the beginning of the twenty-first century, there has been a significant and growing interest in the fields of nanoscience and nanotechnology (Hulla et al. 2015). Nanotechnology can be defined as the science and engineering concerned with the design, synthesis, characterization, and application of materials and devices at the nanometer scale (Saini et al. 2010). Also, nanotechnology is used in many technology and industry fields such as information technology (Chong 2004), homeland security (Reynolds and Hart 2004), transportation (Mathew et al. 2019), environmental science (Taran et al. 2021), energy (Abdin et al. 2013; Ahmadi et al. 2019), food science (Singh et al. 2017), and medicine (Mehta et al. 2008).

On the other hand, nanomedicine is defined as the application of nanotechnology to health according to the European Technology Platform on Nanomedicine. Here, nanomedicine exploits the improved and often novel physical, chemical, and biological properties of materials at the nanometric scale (Boisseau and Loubaton 2011). Thus, nanomedicine products are nanoparticles (NPs) that can be used for imaging (Padmanabhan et al. 2016), targeted cancer therapy (Xu et al. 2019), drug and gene delivery (Zhou et al. 2018), tissue engineering (Fathi-Achachelouei et al. 2019), and theranostics (Kucharczyk et al. 2019). NPs are particles with at least one dimension smaller than one micron (Buzea et al. 2007). Nanoparticular systems, ranging in size from a few nanometers such as micelles to several hundred nanometers, such as liposomes, can easily interact with biomolecules located on both the cell surface and inside (Boisseau and Loubaton 2011).

The nanometer-scale favors the drug delivery application since nanosized formulations have a larger surface to volume ratio than microsized formulations. For instance, less than 0.01% of the injected dose of drugs in the angstrom size typically accumulates in the target region, while the same value is approximately 1–5% for nanoparticles (Wolfram et al. 2015). Hence, the larger surface area of NPs may improve the efficacy of the therapies. Moreover, the distribution, targeting ability, and toxicity of NPs in the body are mediated by their shape and size. According to the literature, approximately 100 nm is the optimum size for NPs to avoid immediate clearance by the lymphatic system (Rizvi and Saleh 2018). However, NPs with a size of 100 nm result in restricted NP accumulation around tumor blood vessels and poor penetration into the tumor parenchyma (Zein et al. 2020; Moghimi et al. 2001). In contrast, NPs smaller than 10 nm are cleared by renal excretion and phagocytosis (Barua and Mitragotri 2014). Nanometer size is also important for passive targeting in cancer because of the enhanced permeability and retention (EPR) effect due to the leaky vasculature of solid tumors and absence of lymphatic drainage (Bertrand et al. 2014; Farjadian et al. 2019).

Furthermore, the surface of NPs can be functionalized with small and larger molecules like biomolecules (e.g. peptides, aptamers, antibodies) via covalent bonds for specific and active targeting. In addition, the surface of NPs can be made more hydrophilic by coating with polymers such as polyethylene glycol (PEG) to reduce the opsonization (Rizvi and Saleh 2018). After intravenous (i.v.) administration, NPs are quickly opsonized and cleared by the macrophages (Yoo et al. 2010). Opsonization is the binding of the opsonins (serum proteins) to the surface of the NPs, which are recognized by the macrophage scavenger receptor and internalized (Li and Huang 2008). These macrophages are known as the reticuloendothelial system (RES), which consists of the liver and spleen, and is the first barrier that removes many NPs from circulation (Zein et al. 2020). Thereby, PEGylation is a strategy often used to increase the circulation times of NPs in the body while diminishing the RES uptake and favoring the target uptake.

**Fig. 1:**
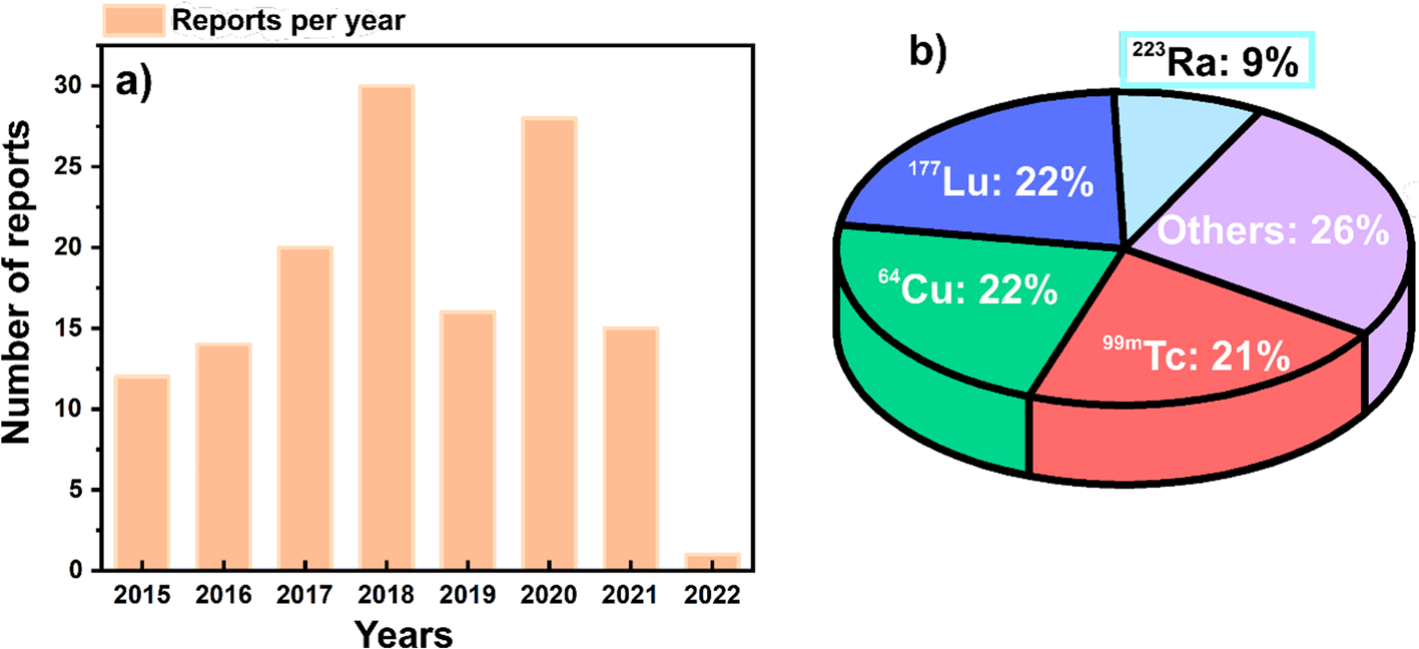
**a** Number of publications per year related to radiolabeled nanomaterials for biomedical applications (2015–2022). **b** Publication percentage of radiolabeled nanomaterials using different radionuclides

The increasing number of publications per year index related to radiolabeled nanomaterials for biomedical applications corroborate the growing interest in the field (Fig. 1a). Tthe radiolabeling of nanomaterials has been performed using different radionuclides, with technetium-99m (^99m^Tc), copper-64 (^64^Cu), lutetium-177 (^177^Lu) being the most popular for this application. However, other radionuclides like radium-223 (^223^Ra) and carbon-14 (^14^C) (Nallathamby et al. 2015; Soubaneh et al. 2020), gallium-68 (^68^Ga) (Biagiotti et al. 2019; Marenco et al. 2021), zirconium-89 (^89^Zr) (Chen et al. 2017, 2018), iodine-125 (^125^I) (Jeon et al. 2016; Tao et al. 2021), yttrium-90 (^90^Y) (Paik et al. 2015), gold-199 (^199^Au) (Zhao et al. 2016a), barium-131 (^131^Ba) (Falco Reissig et al. 2020) etc., have also been used for radioactive-labeling nanomaterials in radiopharmacy (Fig. 1b).

The design of functionalized radiolabeled nanomaterials with specific-target and imaging moieties, showing safety and high circulation times without metabolic degradation, is attractive for Nuclear Medicine, especially for theranostic applications. Theranostics combine diagnostic and therapeutic applications, which contribute to implementing individualized dosimetry-based treatment (Hosono 2010). In fact, the use of radiolabeled NPs has mainly been evaluated in cancer for molecular imaging (Bluemel et al. 2015; Surasi et al. 2015; Jin et al. 2017; Thakare et al. 2019; Du et al. 2017), radionuclide therapy alone (Cai et al. 2017; Cvjetinović et al. 2021), or combined with other therapies such as plasmonic photothermal (González-Ruíz et al. 2018; Mendoza-Nava et al. 2017), chemotherapy (Gibbens-Bandala et al. 2019), and immunotherapy (Pei et al. 2021a), as well as theranostics (Imlimthan et al. 2021). Most of these works use preclinical cancer models.

Molecular imaging combines in vivo imaging and molecular biology in order to identify or describe living biological processes at a cellular and molecular level using noninvasive procedures (Wu and Shu 2018). Positron- and gamma-emitting radiolabeled NPs are used for molecular imaging using positron emission tomography (PET) and single-photon emission computed tomography (SPECT), respectively. These nuclear imaging modalities (PET and SPECT) provide functional information. In addition, imaging studies with radiolabeled NPs usually combine PET and SPECT imaging with computed tomography (CT) to add anatomical information (Wong et al. 2017; Lee et al. 2017). In case the radiolabeled NP is a material with magnetic properties useful for magnetic resonance imaging (MRI), then it can be used as a dual-modal (PET/MRI and SPECT/MRI) molecular imaging probe (Shi and Shen 2018; Gao et al. 2016). Also, MRI provides morphological and anatomical information. Some radiolabeled NPs can be used as three-modal imaging probes. For instance, they combine PET/MRI/fluorescence optical imaging (Thakare et al. 2019; Kim et al. 2018), and PET/MRI/photoacoustic tomography (Jin et al. 2017), adding valuable information. These imaging techniques are based on different basic physical principles. These techniqueshave certain advantages and disadvantages in terms of sensitivity and specificity to contrast agents, tissue contrast, spatial resolution, quantitation, and tissue penetration (Baetke et al. 2015).

Nevertheless, to date, only a few NPs are clinically approved and used to detect sentinel lymph nodes by SPECT imaging after radiolabeling with ^99m^Tc (Thakor et al. 2016). This review paper presents the state-of-the-art NPs labeled with ^64^Cu and ^99m^Tc for PET and SPECT imaging, respectively, combined with CT, MRI, fluorescence optical imaging or photoacoustic tomography. Radionuclide therapy is a safe and effective approach to treat cancer by delivering ionizing radiation using radiopharmaceuticals that either bind preferentially to cancer cells or accumulate by physiological mechanisms (Sgouros et al. 2020). For therapeutic aims, the radiopharmaceuticals are formulated with radionuclides that emit Auger electrons, beta or alpha particles, releasing the ionizing radiation in the proximity of the target. Auger electrons have high linear energy transfer (LET) (4–26 keV/µm) and the shortest range (2–500 nm), limiting their application to treat single cancer cells once the radionuclide had crossed the cell membrane and reached the nucleus (Poty et al. 2018). In contrast, alpha particles are more effective for small neoplasms or micrometastases because of their highest LET (80 keV/µm) and short-range (50–100 µm) (Poty et al. 2018). Conversely, the beta particles are more effective in treating medium to large tumors owing to their longest particle range (0.5–12 mm) and LET (0.2 keV/µm) (Poty et al. 2018). We also present the state-of-the-art of NPs labeled with the beta emitter ^177^Lu and the alpha emitter ^223^Ra for radionuclide therapy (^177^Lu, ^223^Ra) and theranostics (^177^Lu). In addition, the chemical and nuclear properties of the selected radionuclides, radiolabeling of NPs, the EPR effect, and other strategies to improve the efficacy of NPs and their toxicity are overviewed in this review paper. Liver radioembolization using microspheres labeled with the beta emitters ^90^Y or holmium-166 (^166^Ho) is one of the most successful clinical applications using radiolabeled microparticles (D’Abadie et al. 2021). This application is also described in the context of the present review.

## Nanomaterials

**Fig. 2:**
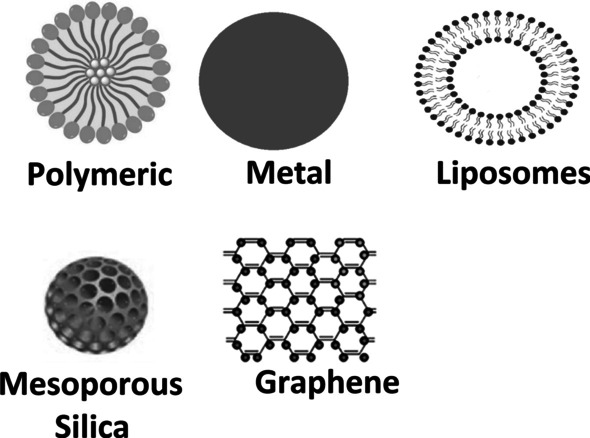
Examples of nanomaterials available or under research worldwide, representing the main forms/structures, including polymeric nanoparticles, metal nanoparticles (gold and silver mainly), liposomes, mesoporous silica, and graphene (and graphene derivatives, like graphene quantum dots and graphene oxide)

Nanomaterials are materials with structural components smaller than one micrometer in at least one dimension (Buzea et al. 2007), which represent a vast class of compounds (Fig. 2). They can be classified into three major categories: (1) inorganic nanomaterials, which comprise noble metals, magnetic metals, quantum dots, and non-metals, (2) organic nanomaterials, which consist of polymers and lipids; and (3) carbon nanomaterials. Inorganic nanomaterials are a multifaceted class that comprises two groups (1) metallic and (2) non-metallic. The development of metallic NPs is of significant interest due to their unique and relevant characteristics, including their optical activity, electrical and magnetic properties, mechanical stability, and large surface area (Khan et al. 2019).

The non-metallic nanomaterials group consists mainly of mesoporous silica, formed by groups of silicon oxide organized in hexagonal, cubic, or lamellar structures (Cong et al. 2018). According to IUPAC (International Union of Pure and-Applied Chemistry), its pores should have a diameter of 2–50 nm (Costa and Paranhos 2020). The interest for this material is related to its distinct characteristics, such as porous structures with adjustable volume and diameter, large surface area, and high density of silanol on the surface, which allows the nanomaterial to function (Vallet-Regí et al. 2007; Gisbert-Garzarán et al. 2020). This nanomaterial has several applications, such as targeting drugs and genes (Aquib et al. 2019; Kesse et al. 2019), antibacterial treatment (Bernardos et al. 2019), and bone tissue regeneration (Kanniyappan et al. 2021).

Mesoporous magnetic silica has a magnetic core comprising iron oxide bound to silica (Fe_3_O_4_–SiO_2_) or hollow mesoporous silica NPs (MSNs) (Wu et al. 2020a). However, the use of hollow MSNs, with a large central hole combined with an external mesoporous silica shell, offers an additional advantage due to the higher loading capacity. They have greater storage capacity and can remain in the tissues for a limited period without causing damage. At the same time, magnetic MSNs allow the targeting of drugs, genes, and imaging agents through an external magnetic field (Kesse et al. 2019). Superparamagnetic materials, having a single-domain character, which causes a phenomenon called superparamagnetism. These kinds of materials do not retain any residual magnetization after removing the external magnetic field, thus preventing possible agglomeration of nanoparticles in the bloodstream and the formation of possible embolism (Lu et al. 2007).

Organic nanomaterials are divided into two major categories (1) organic lipid nanomaterials and (2) organic polymeric nanomaterials. These nanomaterials are mainly used to develop nanoplatforms for targeting drugs, genes, and imaging agents. The structures of liposomes can be obtained from lipid compounds, both of which have the advantage of biocompatibility and easy encapsulation of substances. Liposomes consist of a bilayer of amphiphilic lipids, which have proven to be efficient carriers for targeting various substances since they possess amphipathic domains around an aqueous nucleus and enable the rapid integration of molecules with different physicochemical properties (Penoy et al. 2020). Therefore, hydrophilic substances are encapsulated in the core of the nanostructure, and lipophilic substances are intercalated in the lipid bilayer (Romero-Arrieta et al. 2020). Highly toxic or low bioavailability drugs benefit from the stabilizing nature and improved biodistribution of liposomes and micelles in circulation. Organic lipid nanomaterials and organic polymeric nanomaterials are often synthesized using polymers or coated to avoid recognition by cells and components in the reticuloendothelial system (Moghimi and Reviews 1998; Bobo et al. 2016; Maiolo et al. 2015).

The group of polymeric organic nanomaterials can be divided into two categories: (1) biodegradable polymers and (2) non-biodegradable polymers. They can be obtained in different morphologies of nanosystems, such as nanospheres (it has a polymeric matrix nucleus), nanocapsules (composed of a polymeric shell containing an oily or aqueous nucleus), and dendrimers (formed by a branching nucleus). Additionally, polymeric nanosystems are capable of releasing drugs in a controlled, and sustained manner in the body through three mechanisms: (1) the active molecules cross the polymer barrier by diffusion; (2) erosion of the polymeric material, and (3) penetration of solvent/swelling of the system (Martins et al. 2018). Among the polymers, biodegradable polymers are the most interesting and used because of their intrinsic properties, such as biodegradability (Jana et al. 2020), biocompatibility (Biswas et al. 2020), colloidal stability (García et al. 2020), non-inflammatory (García-Valdivia et al. 2020), and non-immunogenic nature (Andorko et al. 2016), including their small size, functionalizable surface, and good solubility (Carvalho and Conte Junior 2020). Biodegradable polymers are degraded in vivo, preferably by hydrolysis or enzymatic breakdown, producing biocompatible and non-toxic by-products eliminated by normal metabolic pathways (Mir et al. 2017).

Composite nanomaterials combine a number of properties of all the previously listed groups. These are often systems composed of metallic or metallic-oxide materials coated with a silica or polymer corona which can be further chemically modified (Novy et al. 2020). The motivation for preparing such a composite nano-construction is the combination of the most favorable properties of the types mentioned above of nanomaterials to be used as multimodal theranostic nanoprobes. By combining it with various novel nanoparticle-based activatable probes, molecular imaging technologies can provide a feasible approach to visualize tumor-associated microenvironment parameters noninvasively and realize the accurate treatment of tumors.

Furthermore, graphene and its derivatives like graphene quantum dots and graphene oxide are carbon-based nanomaterials. Graphene is a crystalline material and a two-dimensional nanostructure with sp2 hybridized carbon atoms that form a hexagonal honeycomb structure (Magne et al. 2021a). The graphene surface can interact with other molecules through physical adsorption mechanisms (π-π interactions), or chemical interaction (covalent bonding). For this, the structure of graphene is previously modified through the introduction of defects or functional groups such as carboxyl, carbonyl and amino (Felix et al. 2021). Several biomedical applications of graphene and its derivatives have been reported so far, which were recently reviewed by our group (Magne et al. 2021a).

Nanoparticles can achieve a diagnostic and, at the same time, therapeutic effect depending on the type of radionuclide and/or chemical modification enabling controlled drug release. The chemical behavior of nanoparticles labeling depends on the category mentioned above (i–iiii). In general, labeling of the prepared nanoparticles might be performed (a) by surface sorption of the radionuclide to the surface of the nanoparticle directly, (b) intrinsic encapsulation of the radionuclide into the core of the nanoparticle during the synthesis, (c) chelation of radionuclide by ligands (mostly polydentate, e.g., DTPA (Diethylenetriamine pentaacetate), DOTA (1,4,7,10-tetraazacyclododecane-1,4,7,10-tetraacetic acid), and NOTA (1,4,7-triazacyclononane-N,N′,N″-trisacetic acid) derived analogs) directly attached or linker spaced on the surface of the nanoparticle.

Parameters like particle size, specific surface area, contact time, and temperature play an important role. Moreover, the liquid phase composition, such as pH, the concentration of radionuclide ions, ionic strength, the presence of complexation ligands, etc., should be considered (Suchánková et al. 2020a). Same conditions should be followed during intrinsic labeling, and also reaction conditions of nanoparticles preparation must be considered.

## The EPR effect and other strategies to improve the efficacy of nanoparticles

The natural accumulation of NPs after i.v. administration is in the liver, which can negatively affect their targeting. Suppression of this effect leads to a better uptake in targeted tissues as well as a decrease of radiation burden to surrounding tissues. Proper targeting of NPs can generally be achieved either by binding system stabilizers/targeting vectors to the surface of the NPs (antibodies, polymers, peptides, etc.) or by using the EPR effect (Ballinger 2018; Pratt et al. 2016; Sharma et al. 2021).

**Fig. 3:**
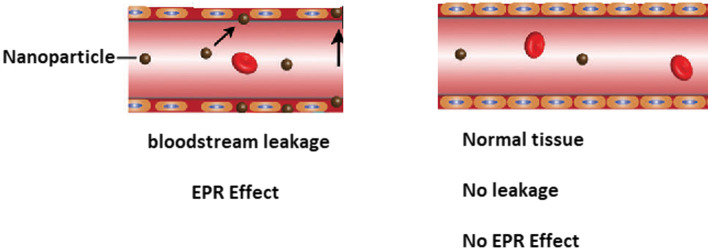
Representation of the EPR effect demonstrating the leakage of the nanoparticles from the bloodstream by the fenestrations in the blood vessels caused by the unorganized tissue

The EPR effect is a phenomenon (Fig. 3), which occurs in solid tumors sites due to their anatomical and pathophysiological differences from normal tissues. The exacerbated angiogenesis promoted by the uncontrolled cell proliferation during cancer leads to high vascular density in solid tumors. The new vasculature produced during this angiogenesis process has large gaps between endothelial cells, which cause the extravasation of nanoparticles into the lumen of the tumor (permeation effect). Also, the new vasculature grows in a distorted form, causing a deficiency in the lymphatic drainage, leading to permanent retention of the nanoparticle in the tumor (retention effect). Although the EPR effect is the most well-known effect related to nanoparticle efficacy, it is not the only process involved in the mechanism (Shi et al. 2020; Yhee et al. 2013). Recently, it was detected that immune cells in the tumor microenvironment play important roles in accumulation, retention, and intratumoral distribution. For instance, Korangath et al. (Korangath et al. 2020) showed that NPs were retained in the tumor by association with dendritic cells, neutrophils, monocytes, and macrophages and not just by the EPR effect.

Also, the use of active targeting is a good strategy to improve tumor accumulation, preventing nanoparticle dispersion on non-primary targets. In this direction, the use of ligands like fragments of antibodies, monoclonal antibodies, aptamers, and peptides represent an interesting approach.

## Radiolabeled nanomaterials for molecular imaging

### ^***99m***^***Tc-based radiolabeled nanomaterials***

^99m^Tc has a half-life of 6 h and emits gamma rays of 140.5 keV. ^99m^Tc is available worldwide due to its cheap production using the ^99^Mo/^99m^Tc radionuclide generator. In the generator system, ^99^Mo transforms to ^99m^Tc at a rate of 87% and to ^99^Tc at a rate of 13% by beta decay with 740–780 keV energy. Tc has eight oxidation states from − 1 to + 7, being + 7 and + 4 the most stable valency. Its + 7 valence state (^99m^TcO_4_^−^) does not combine directly with other compounds. Since ^99m^TcO_4_^−^ is chemically nonreactive and cannot label any compound by direct addition, radionuclide reduction to lower oxidation states is required. The reduction is obtained by various reducing agents include stannous chloride, stannous citrate, stannous tartrate, sodium borohydride, ferrous sulfate, etc. (Saptiama et al. 2016; Hou et al. 2007; Hasan and Prelas 2020).

The use of ^99m^Tc has continued to evolve, especially with modern gamma cameras with advanced electronics and computing systems, revolutionizing nuclear medicine procedures. This development process continued until the first ready-to-use lyophilized kit for radiolabeling with ^99m^Tc in the 1970s. Many new radiopharmaceuticals have been prepared with the discovery of very easy-to-use defined as shake and bake kits (Saleh 2011).

The kits are optimized to ensure that the desired complex has a high labeling yield. Several factors have influenced the labeling yield and the stability of the complex, such as the amount of reducing agent and ligand, pH, and temperature. The chemical groups suitable for direct radiolabeling by chelating technetium radionuclide are –OH, –COOH, –C=O, –PO_4_, –P_2_O_7_-, –NH_2_, –SOOH, –SOONH, –SOONH_2_, –OCH_3_. By using these chemical groups, radiolabeling can be done directly with ^99m^Tc and through different chelate groups. In this context, the chelators frequently used with ^99m^Tc are DOTA and DTPA of small molecules, colloids, and polymeric NPs. At the same time, the chelators that are frequently used with ^99m^Tc labeled lipid-based NPs (such as; micelles, liposomes, solid LNPs) are HMPAO (D,L-hexamethylene-propyleneamine oxime).

To date, a few ^99m^Tc-labeled NPs, mainly colloids, are clinically approved (Table 1). On the other hand, iron oxide NPs, oligomeric NPs, gold nanoparticles (AuNPs), micelles, liposomes, solid lipid nanoparticles (SLNs), MSNs have been ^99m^Tc-labeled with/without chelate agents and evaluated in different preclinical cancer models as shown in Table 2.

**Table 1:** Clinically approved ^99m^Tc-labeled nanoparticles and microparticles by SPECT imaging

Type	Trade name	Particle size	Evaluated applications	References
Sulfur colloid	Technecoll (US)	100–300 nm	Lymph node, bone marrow, GI, liver, and spleen imaging	Thakor et al. 2016; Palestro et al. 2006)
Albumin colloid	Nanocoll (EU)	6–80 nm	Lymph node, inflammation, melanoma, and prostate imaging	Thakor et al. 2016; Gommans et al. 2009)
SnF_2_ colloid	Hepatate (France)	< 200 nm	Lymph node, GI, liver, and spleen imaging	Thakor et al. 2016; McClelland et al. 2003)
Re_2_S_7_ colloid	Nanocis (EU)	10–70 nm	Lymph node, GI, melanoma, and prostate imaging	Thakor et al. 2016; Tsopelas 2014)
Albumin colloid	Senti-Scint	100–600 nm	Lymph node imaging of breast	Thakor et al. 2016; Kim et al. 2001)
Tilmanocept	Lymphoseek	7 nm	Lymphatic mapping and sentinel lymph node localization	Surasi et al. 2015)
Albumin macroaggregates	Macroaggregated albumin (MAA)	10–90 microns	Lung perfusion imaging	Hunt et al. 2006; Hung et al. 2000)

**Table 2:** Representative studies evaluating ^99m^Tc-labeled nanoparticles in preclinical cancer models

^99m^Tc-labeled NPs	NPs/Chelate	Experimental conditions t(min)/T(ºC)/pH	Radiochemical yield (%)	Evaluated applications	References
[^99m^Tc]Tc-IO-NPs-RGD	IO-NPs/cRGDfK-Orn3-CGG	30 min/25 °C/pH 8	> 98%	Molecular imaging of ανβ3-mediated tumor expression and feasibility for hyperthermia treatment. In vitro and in vivo results	Tsiapa et al. 2014)
[^99m^Tc]Tc-NPs-FA	Oligomeric FA-NPs/chelate-free	30 min/25 °C	–	Cell uptake by folate receptor-positive tumor targeting. In vitro results on HepG2 tumor cells	Liang et al. 2018)
[^99m^Tc]Tc-EDDA/HYNIC-GGC-AuNP-mannose	AuNPs/EDDA-HYNIC	20 min/100 °C	> 95%	Biodistribution and microSPECT/CT images of sentinel lymph node detection. In vivo results in Wistar rats	Ocampo-García et al. 2011)
[^99m^Tc]Tc-{(Au^0^)_200_-G5.NHAc-DOTA-FI-*m*PEG-(PEG-duramycin)} DENPs	Au-DENPs/DOTA	30 min/25 °C	99%	SPECT/CT imaging of chemotherapy-induced tumor apoptosis. In vitro and in vivo results	Xing et al. 2018)
[^99m^Tc]Tc-citrate-AuNPs	AuNPS/chelate free	–	95.20 ± 2.70%	Biodistribution patterns. In vivo results on solid tumor-bearing mice	Essa et al. 2020)
[^99m^Tc]Tc-PC:PEG2000-DSPE:SDC:DTPA-PE	Micelles/DTPA-PE	30 min/25 °C	87 ± 1.21%	Potential radiotracers for detection of infection/inflammation. In vitro results against *S. aureus* and *E.coli*	Silindir-Gunay and Ozer 2020)
[^99m^Tc]Tc-DSPE-PEG2000-DTPA PM	Polimeric micelles/DTPA	15 min/25 °C/pH 7	93.8 ± 2.1%	Biodistribution and scintigraphic images. In vitro and in vivo results on 4T1 tumor-bearing mice	Oda et al. 2017)
[^99m^Tc]Tc-HMPAO-blue-biotin-liposome	Liposome/HMPAO	30 min/25 °C	92.1 ± 1.9%	Scintigrams of the sentinel lymph node. In vivo results	Phillips et al. 2001)
[^99m^Tc]Tc-HMPAO-liposome	Liposome/HMPAO	30 min	91%	Tumor imaging with gamma camera. In vivo results	Goins et al. 1994)
[^99m^Tc]Tc-liposome	Liposome/chelate-free	20 min/25 °C	> 95%	Biodistribution and scintigraphic images. In vivo results on CD1 mouse bearing breast cancer	Navarro et al. 2011)
[^99m^Tc]Tc-SpHL-DTPA-folate-PTX (folate-coated long-circulating and pH-sensitive liposomes) [^99m^Tc]Tc-SpHL-DTPA-PTX	Liposome/DTPA Liposome/DTPA	15 min/25 °C/pH 7.4 15 min/25 °C/pH 7.4	98% 98.4 ± 1.1%	Biodistribution and scintigraphic images. In vivo results on BALB/c nude mice bearing breast cancer	Monteiro et al. 2018)
[^99m^Tc]Tc-oxine-SLNs [^99m^Tc]Tc-oxine-CH-SLNs	SLN/oxine SLN/oxine	1 h/25 °C 1 h/25 °C	100% 100%	Biodistribution patterns. In vivo results on BALB/c mice	Gharibkandi et al. 2019)
[^99m^Tc]Tc-PEG-MnOx-MSNs	MSNs/chelate-free	60 min/37 °C/pH 7.0	99.1 ± 0.6%	SPECT-MRI dual-modal imaging (nanotheranostics). In vitro and in vivo results on tumour-bearing mice	Gao et al. 2016)
[^99m^Tc]Tc-DTPA-MSNs	MSN/DTPA	5 min/25 °C/pH 7.0	98.3 ± 0.7%	Biodistribution and scintigraphic images. In vivo results on healthy Swiss mice	Barros et al. 2015)

### ^***64***^***Cu-based radiolabeled nanomaterials***

Among the Cu radioisotopes, ^64^Cu is the most studied for biomedical applications using PET due to its attractive nuclear qualities. It decays by electron capture (41%, 1346 keV), positron (19%; 657 keV) and beta (40%; 578.7 keV) emissions, with an average tissue penetration of 0.7 and 0.95 mm for positron and beta particles, respectively (Ahmedova et al. 2018). Its relatively long half-life of 12.7 h allows for shipping to distant centers and for longer in vivo imaging studies compared to the well-established PET radionuclides: fluor-18 (109.7 min), gallium-68 (67.7 min), and carbon-11 (20.4 min). The low positron energy of ^64^Cu is closer to the positron energy of fluor-18 (634 keV), which favors image resolution (Conti and Eriksson 2016). Besides, the beta particles and Auger electrons emitted from the electron capture decay are useful for radionuclide therapy. In particular, the Auger electrons have a very low average energy (2 keV) and average tissue penetration (∼126 nm), resulting in high LET radiation that is potentially killing cancer cells (McMillan et al. 2015). Additionally, ^64^Cu can be produced in reactors and cyclotrons. The most common method is currently through proton irradiation of enriched nickel-64 solid target [^64^Ni(p,n)^64^Cu] in small medical cyclotrons, achieving the highest yields in the proton energy range of 10–15 MeV and enough high purity product (Synowiecki et al. 2018). In nuclear reactors, ^64^Cu can be produced by ^63^Cu(n,γ)^64^Cu and ^64^Zn(n,p)^64^Cu reactions using thermal and fast neutrons, respectively, with correspondingly low and high specific activities (Niccoli Asabella et al. 2014). However, the use of the high-specific activity ^64^Zn(n,p)^64^Cu reaction is limited because of the co-production of the zinc-65 radioisotope with a half-life of 245 days (Shokeen and Anderson 2009).

Cu’s most common oxidation states are 1+ and 2+, where ionic radius are 77 and 73 pm, respectively. Cu^+^ forms complexes without any crystal-field stabilization energy are not recommended for incorporation into radiopharmaceuticals due to insufficient kinetic stability. At the same time, Cu^2+^ is the best option for radiopharmaceutical applications owing to less labile toward ligand exchange by the presence of some crystal-field stabilization energy (Wadas et al. 2007). Moreover, Cu^+^ coordination compounds have been reported by complexation with N/N-, and phosphine-donor ligands, whereas Cu^2+^ coordination compounds are formed by complexation with N–, O– and S–, N– and O–, N– and S–, N/N–, and S/S– donor ligands (Krasnovskaya et al. 2020).

^64^Cu-labeled NPs are promising for cancer imaging by PET in combination or not with MRI or optical imaging. ^64^Cu-chelate complexation, chelate-free conjugation, and neutron activation are the main approaches used for ^64^Cu radiolabeling of NPs so far. DOTA, NOTA, NODAGA (1,4,7-triazacyclononane, 1-glutaric acid-4,7-diacetic acid) and 4-DEAP-ATSC (diacetyl 4,4′-bis(3-(N,N-diethylamino)propyl)thiosemicarbazone) have been the most used chelates for radiolabeling NPs. The best yields (> 95%) were obtained using NOTA/NODAGA chelates, performing the ^64^Cu-chelate complexation at the last step of the radiopharmaceutical preparation, except for ultra-pH sensitive (UPS) polymer (Huang et al. 2020) and micelles (Paiva et al. 2020) NPs. Conversely, several NPs have been ^64^Cu-labeled by chelate-free conjugation method with yields between 75 and 97% after reacting at 25–37 °C for 10–60 min and pH 5.5–7 (Jin et al. 2017; Madru et al. 2018; Xu et al. 2018; He et al. 2021). In particular, the copper sulfide ([^64^Cu]CuS) NPs were prepared with > 98% yield by doping CuS at pH 9 and heating at 65–90 °C for 15 min before functionalization for specific tumor targeting (Cui et al. 2018; Cai et al. 2018). Additionally, neutron activation is another method used for ^64^Cu radiolabeling NPs, delivering a radio-nanoprobe with good stability for cancer-targeted, controlled drug delivery and PET imaging (Oliveira Freitas et al. 2017).

Liposomes, lipid nanoparticles (LNPs), lipid nanodiscs (LND), micelles, UPS polymers, carbon quantum dots (CQDs), polyglucose nanoparticles (Macrin), melanin, gadolinium nanoparticles (AGuIX), silicon, silica gadolinium nanoparticles (SiGdNPs), iron-gallic acid coordination nanoparticles (Fe-GA-CPNs), superparamagnetic manganese ferrite (MnFe_2_O_4_), and CuS nanoparticles have been ^64^Cu-labeled and evaluated as PET tracers in different preclinical cancer models as shown by Table 3. Also, it has been shown that functionalization of these ^64^Cu-labeled NPs with peptides, programmed cell death-1 (PD-1) antibody, or anti-PSMA site-specific cysteine-diabody (cys-DB) enhanced tumor uptake. In particular, the radiolabeled Fe-GA-CPNs (Jin et al. 2017), MnFe_2_O_4_ (Shi and Shen 2018), SiGdNP (Tran et al. 2018), and AGuIX (Thakare et al. 2019) exhibited favorable outcomes for PET/MRI dual imaging of tumors. Among them, the ^64^Cu-labeled Fe-GA CPNs after surface modification with the hydrophilic polymer PEG exhibited much more efficient passive tumor accumulation (EPR effect) upon intravenous administration into tumor-bearing mice (Jin et al. 2017). Moreover, the introduction of the near-infrared heptamethine cyanine dye IR783 allowed obtaining nanorradiopharmaceuticals for PET/MRI/optical imaging (Thakare et al. 2019). Furthermore, doxorubicin (DOX)-loaded ^64^Cu-labeled NPs showed favorable results for chemotherapy and PET imaging (Du et al. 2017; Wong et al. 2017). Thereby, the preclinical reports of ^64^Cu-labeled NPs are mainly focused on their use for treatment planning and monitoring the therapeutic responses by PET imaging.

**Table 3:** Representative studies evaluating ^64^Cu-labeled nanoparticles in preclinical cancer models

^64^Cu-labeled NPs	NPs/Chelate	Experimental contitions t (min)/T(ºC)/pH	Radiochemical yield (%)	Evaluated applications	References
[^64^Cu]Cu-DOX-anti-PD-1-Liposomes [^64^Cu]Cu-PEG-Liposomes (MM-DX-929)	Liposomes/DOTA Liposomes/4-DEAP-ATSC	2 h/43 °C/pH 6.5 1 min/25 °C/pH 6	62% > 90%	PET imaging of PD-1-overexpressing breast tumors. In vitro and in vivo results of enhanced chemotherapy effects PET imaging of breast tumors	Du et al. 2017) Lee et al. 2018)
[^64^Cu]Cu-AGuIX [^64^Cu]Cu-IR783 –AguIX	AGuIX/DOTA AGuIX/NODAGA	1 h/37 °C/pH 5.5 45 min/37 °C/pH 5.5	> 98% –	PET imaging of liver cancer. Positive i*n vivo* results of radionuclide therapy with AGuIX after irradiation using an X-ray source PET/MRI/ optical imaging of TSA tumors	Hu et al. 2017) Thakare et al. 2019)
[^64^Cu]Cu-DOX-PEG-LNP [^64^Cu]Cu- cys-DB- PEG-LNP [^64^Cu]Cu-anti-CEA- PEG-DBCO LND	LNP/DOTA LND/DOTA	45 min/43 °C/pH 5.5 –	> 75% 70%	PET imaging of prostate cancer. In vivo results of enhanced chemotherapy effects PET imaging by targeting carcinoembryonic antigen (CEA) in breast cancer	Wong et al. 2017) Wong et al. 2020)
[^64^Cu]Cu-PEG-Fe-GA-CPNs	Fe-GA-CPNs/chelate-free	60 min/37 °C/pH 5.5	75%	PET imaging and photoacoustic tomography/MRI of breast cancer. In vitro and in vivo results of photothermal therapy	Jin et al. 2017)
[^64^Cu]Cu-Macrin	Macrin/NODAGA	30 min/90 °C/pH 6	> 99%	PET and optical imaging of tumor-associated macrophages in lung carcinoma	Kim et al. 2018)
[^64^Cu]Cu-SiGdNP	SiGdNP/NODAGA	30 min/37 °C/pH 5.8	No reported	PET/MRI dual imaging of metastatic mammary adenocarcinoma (TS/A)	Tran et al. 2018)
[^64^Cu]Cu-PEG-dopamine-RGD-MnFe_2_O_4_	MnFe_2_O_4_/DOTA	40 min/50 °C/pH 6.5	65%	PET/MRI dual imaging by targeting integrin α(v)β(3) in glioblastoma	Shi and Shen 2018)
[^64^Cu]Cu-silicon [^64^Cu]Cu-CQDs	Silicon and CQDs /NOTA	30 min/25 °C/pH 6	> 99%	PET imaging of epidermoid carcinoma	Licciardello et al. 2018)
[^64^Cu]CuS-PEG-RGD [^64^Cu]CuS-PEG-bombesin [^64^Cu]CuS-PEG	CuS/chelate-free CuS/chelate-free CuS/chelate-free	15 min/95 °C 15 min/65 °C/pH 9 15 min/95 °C	> 98% > 98%	PET imaging by targeting integrin α(v)β(3) in glioblastoma. In vitro and in vivo results of photothermal therapy PET imaging by GRPr in prostate cancer PET imaging of melanoma and ovarian cancer. In vitro and in vivo results of photothermal therapy combined with immunotherapy	Cui et al. 2018) Cai et al. 2018) Cao et al. 2020)
[^64^Cu]Cu-GE11-micelles	Micelles/NOTA	15 min/37 °C/pH 5.5	23%	PET imaging by targeting the epidermal growth factor receptor in colon cancer	Paiva et al. 2020)
[^64^Cu]Cu-PEG- melanin	Melanin/chelate-free	1 h/40 °C/pH 5.5	–	PET imaging of epidermoid carcinoma. In vivo results of radionuclide therapy	Zhou et al. 2020)
[^64^Cu]Cu-UPS polymers	UPS polymers/NOTA	15 min/37 °C/pH 6.5	> 95%	PET imaging of small occult tumors in the brain, head, neck and breast of mice by targeting tumor-acidosis	Huang et al. 2020)
[^64^Cu]Cu-PEG-PPa-Trp2	Trp2 peptide-coassembled NPs /chelate-free	30 min/25 °C	> 97%	PET imaging of melanoma. In vivo results of dendritic cell-based immunotherapy	He et al. 2021)

Additionally, clinical PET images with ^64^Cu-labeled NPs were also reported, for instance, to quantify the variability of the EPR effect of NPs in relation to treatment response in patients with HER2-positive metastatic breast cancer. The authors used the [^64^Cu]Cu-MM-302 nanoprobe prepared by ^64^Cu-chelate (4-DEAP-ATSC) complexation before reaction with MM-302 (HER2-targeted PEGylated liposomal DOX) NPs. [^64^Cu]Cu-MM-302 was safe and stable in patients within the image acquisition time frame. PET/CT imaging showed significant tumor accumulation in bone and brain lesions with high [^64^Cu]Cu-MM-302 deposition at 24–48 h and significant background uptake in the liver and spleen as well (Lee et al. 2017).

Beyond PET imaging, however, there is a lack of reports evaluating the potential of ^64^Cu-labeled NPs for radionuclide therapy, taking into account the beta particles and Auger electrons emitted by ^64^Cu. We only found [^64^Cu]Cu-PEG-melanin NPs evaluated for PET imaging and radionuclide therapy in the reviewed period with promissory results. The authors reached radiolabeled melanin with good stability using the chelate-free conjugation method due to the inherent chelating ability of melanin to [^64^Cu]Cu^2+^ ion. PET images with [^64^Cu]Cu-PEG-melanin exhibited the highest tumor uptake at 4 and 8 h after tail vein injection in epidermoid carcinoma tumor-bearing mice. Tumor growth was significantly decreased compared to control groups at one week after a single intravenous injection of [^64^Cu]Cu-PEG-melanin (~ 55.5 MBq) when tumors reached diameters of 5–8 mm, without radioactive cytotoxicity to normal tissues (Zhou et al. 2020). Therefore, we encourage to continue assessing the efficiency of ^64^Cu-labeled NPs for radionuclide therapy as theranostic agents, also considering nanomaterials’ favorable properties for enhancing targeted radionuclide delivery and retention into tumors.

## Radiolabeled nanomaterials for radionuclide therapy

### ^***177***^***Lu-based radiolabeled nanomaterials***

Among the artificial radioisotopes, ^177^Lu is the most known and routinely used in nuclear medicine. ^177^Lu is a theranostic radioisotope because of its beta and gamma decay. Its low-energy beta particles (mean energy of 134 keV; maximum energy of 498 keV (79%)) have a mean range of 0.7 mm and a maximum of 2.1 mm in soft tissue (Ahmadzadehfar et al. 2020). Furthermore, its emitted photons of 113 keV (6.2%) and 208 keV (10.4%) are useful for ^177^Lu SPECT dosimetry (Müller et al. 2017; Alnaaimi et al. 2021). Moreover, ^177^Lu has a half-life of 6.65 days, which is suitable for radionuclide therapy. On the other hand, ^177^Lu is mainly produced in nuclear reactors with high specific activity through neutron irradiation of either enriched ^176^Lu or ^176^Yb nuclides using lutetium oxide (Lu_2_O_3_) or ytterbium oxide (Yb_2_O_3_) as target material (Talip et al. 2020).

Lu forms complexes with organic ligands of high coordination numbers (6, 7, 8, and 9). DOTA is the macrocyclic ligand most used for [^177^Lu]Lu^3+^ complexation because of its high stability constant (Banerjee et al. 2015). [^177^Lu]Lu^3+^ complexes formation with macrocyclic ligands is very slow when low ligand concentrations are employed. However, at high pH (> 6), insoluble lanthanide hydroxides are formed (Banerjee et al. 2015). Therefore, heating (95–100 °C for 30–40 min) and pH (4.5–6) are critical variables to achieve near quantitative labeling yields of ^177^Lu-labeled peptides (Sharifi et al. 2018; Jowanaridhi and Sriwiang 2019).

^177^Lu-labeled NPs have improved cancer treatment outcomes in preclinical settings by enhancing the radionuclide delivery in tumors. Besides, the use of these labeled NPs in combination or not with other therapies such as chemotherapy, immunotherapy, or plasmonic–photothermal therapy is a unique nanoprobe assessed in preclinical cancer models (González-Ruíz et al. 2018; Gibbens-Bandala et al. 2019; Imlimthan et al. 2021; Pei et al. 2021b). Most of these NPs were radiolabeled by chelate complexation in the last step of the pharmaceutical preparation. Also, the principal conditions for this final step are a narrow range of temperatures (37–95 °C), labeling times (20–60 min), and pH values between 4 and 5 (Tao et al. 2021; Mendoza-Nava et al. 2017; Gibbens-Bandala et al. 2019; Pei et al. 2021b; Vats et al. 2018; Cytryniak et al. 2020*).* The best radiochemical yields (> 95%) were obtained by ^177^Lu-DOTA complexation at the conditions: pH 5 and 30 min @ 90–95 °C (Mendoza-Nava et al. 2017; Cytryniak et al. 2020).

Some studies reported the ^177^Lu-DOTA complexation previous to NPs functionalization (Cai et al. 2017; Imlimthan et al. 2021; González-Ruíz et al. 2017*).* In other cases, radiolabeling by neutron activation of NPs before functionalization (Ancira-Cortez et al. 2020, 2021) and radiolabeling without chelate complexation have been used (Cvjetinović et al. 2021; Ognjanović et al. 2019; Gaikwad et al. 2021). In addition, it was demonstrated that the radiolabeling without chelate complexation approach delivered the ^177^Lu-labeled NPs in high yields (> 98%) after the incubation of these NPs at room temperature for 30 min at pH 5–6. Also, the possible formation of a complex between [^177^Lu]Lu^3+^ and negatively charged carboxylate, hydroxyl and phosphate groups available on coated nanoparticles was proposed as a potential interaction mechanism (Cvjetinović et al. 2021; Ognjanović et al. 2019). In addition, both ^177^Lu radiolabeling via a chelator and direct labeling provided ^177^Lu-labeled NPs with good stability (> 95%) after 24 h (González-Ruíz et al. 2017), 72 h (Cvjetinović et al. 2021), and 96 h (Imlimthan et al. 2021; Ognjanović et al. 2019) of incubation in human serum at 37 °C. ^177^Lu incorporation by replacing a tracer quantity of Eu^3+^ in the EuDPA complex was another radiolabeling method reported (Viana et al. 2020). However, with this approach long reaction times (5 h) and several purification steps are necessary.

Dendrimers (DN), lipidic cubic-phase nanoparticles (cubosomes), chitosan (CH), liposomes, carbon nanospheres (CNS), nanoscale metal–organic frameworks (nMOFs), cellulose nanocrystals (CNC), gold nanoclusters (AuNCs), rare sesquioxides (Lu_2_O_3_), and AuNPs have been ^177^Lu-labeled and evaluated for cancer therapy in different preclinical cancer models as shown by Table 4. Most of them were functionalized with peptides, aptamers, antibodies, glucose, or human serum albumin (HSA) protein for targeted radionuclide therapy. Also, some NPs have been used for the encapsulating of paclitaxel (PTX), doxorubicin (DOX), and vemurafenib (V) to combine chemotherapy and radionuclide therapy in the same nanoprobe. Unfortunately, reports about their clinical application in patients have not been found yet, to the best of our knowledge.

**Table 4:** Representative studies evaluating ^177^Lu-labeled nanoparticles in preclinical cancer models

^177^Lu-labeled NPs	NPs/chelate	Experimental conditions t (min)/T(ºC)/pH	Radiochemical yield (%)	Evaluated applications	References
[^177^Lu]Lu-DNAuNPs-folate-bombesin	AuNPs/DOTA	30 min/90 °C/pH 5	–	Plasmonic–photothermal therapy, optical imaging, and radionuclide therapy by targeting both GRPr and FR overexpressed on breast cancer. In vitro results	Mendoza-Nava et al. 2017)
[^177^Lu]Lu-AuNPs-PEG-Trastuzumab	AuNPs/DOTA	30 min/80 °C/pH 4.5	–	Radionuclide therapy by targeting HER2 overexpressed on breast cancer. In vitro and in vivo results	Cai et al. 2017)
[^177^Lu]Lu-AuNPs-RGD-NLS-Aptamer	AuNPs/DOTA	30 min/90 °C/pH 5	–	Antiangiogenic properties, photothermal therapy, and radionuclide therapy by targeting both α(v)β(3) integrin and VEGF overexpressed in the tumor neovasculature In vitro and in vivo results using rat glioma cell lines	González-Ruíz et al. 2018; González-Ruíz et al. 2017)
[^177^Lu]Lu-CNS-cNGR	CNS/DOTA	20 min/80 °C/pH 4	80 ± 2%	Radionuclide therapy by targeting aminopeptidase N receptors overexpressed on tumor angiogenic blood vessels and tumor cells. In vitro and in vivo results using melanoma cell lines	Vats et al. 2018)
[^177^Lu]Lu-DN(PTX)-Bombesin	DN/DOTA	60 min/37 °C/pH 5	–	Chemotherapy, nuclear imaging, and radionuclide therapy by GRPr overexpressed on breast cancer. In vitro and in vivo results	Gibbens-Bandala et al. 2019)
[^177^Lu]Lu_2_O_3_-HSA	Lu_2_O_3_/chelate-free	30 min/25 °C	84–87%	Radionuclide therapy targeting tumor vasculature. In vitro and in vivo results using melanoma cell lines	Chakravarty et al. 2020)
[^177^Lu]Lu-Cubosome(DOX)	Cubosome/DOTAGA	30 min/95 °C/pH 5	> 99%	Chemotherapy and radionuclide therapy. In vitro results using human-derived HeLa cancer cells	Cytryniak et al. 2020)
[^177^Lu]Lu_2_O_3_-iPSMA	Lu_2_O_3_/chelate-free	Neutron activation at a neutron flux of 1 × 10^13^ n·s^−1^.cm^−2^ for 20 h	–	Optical imaging and radionuclide therapy by targeting prostate-specific membrane antigen (PSMA). In vitro results using PSMA-positive hepatocellular carcinoma cell lines	Ancira-Cortez et al. 2020)
[^177^Lu]Lu@AuNCs	AuNCs/glutathione	20 min/37 °C	901%	Radio-immunotherapy of cancer. In vitro and in vivo results using breast and colon cancer cell lines	Pei et al. 2021b)
[^177^Lu]Lu-PCN-PEG	nMOFs/porphyrin	30 min/37 °C	94%	Radionuclide therapy. In vitro and in vivo results using breast cancer cell lines	Tao et al. 2021)
[^177^Lu]Lu-CH	CH/chelate-free	30 min/25 °C/pH 5	–	Radionuclide therapy. In vitro results using epithelial lung cancer cell lines	Gaikwad et al. 2021)
[^177^Lu]Lu-GML (glucose-modified liposomes)	Liposomes/chelate-free	30 min/25 °C/pH 5.5	97%	Radionuclide therapy by targeting glucose transporters on the tumor vascular endothelium and tumor cells. In vivo results using colon cancer cell lines	Cvjetinović et al. 2021)
[^177^Lu]Lu-CNC-V	CNC/DOTA	60 min/100 °C/pH 4	74 ± 2%	Chemotherapy and radionuclide therapy by targeting the serine/threonine protein kinase BRAF in melanoma. In vitro and in vivo results using a lung metastatic melanoma model	Imlimthan et al. 2021)

**Fig. 4:**
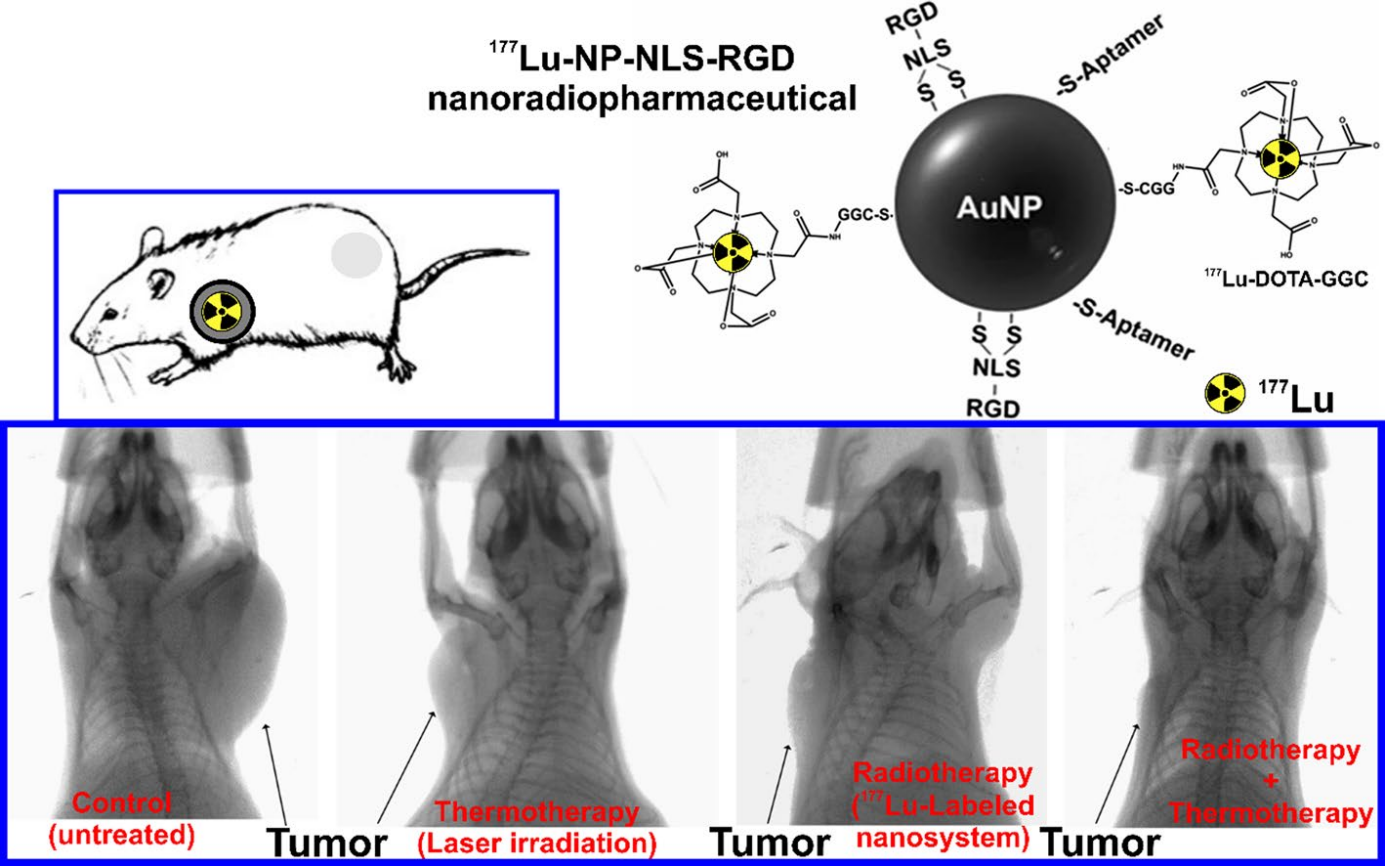
Schematic representation of [^177^Lu]Lu-AuNPs-NLS-RGD-Aptamer nano-radiopharmaceutical. X-ray images (X-Treme/preclinical equipment) of mice with U87MG tumors under thermotherapy (AuNPs-NLS-RGD-Aptamer under laser irradiation), targeted radiotherapy ([^177^Lu]Lu-AuNPs-NLS-RGD-Aptamer), and thermotherapy plus radiotherapy ([^177^Lu]Lu-AuNPs-NLS-RGD-Aptamer under laser irradiation) treatments at 96 h after the last injection (at 25 days of treatment) (González-Ruíz et al. 2018, 2017)

Most of the ^177^Lu-labeled NPs have been prepared using gold nanoparticles. Z. Cai et al*.* (2017) prepared [^177^Lu]Lu-AuNPs-PEG-Trastuzumab nanoconjugate for studying its therapeutic effects in breast cancer by inhibiting the human epidermal growth factor receptor 2 (HER2) (Du et al. 2017). In this study, modified AuNPs with PEG linked to DOTA chelate (for radiolabeling) or to trastuzumab (an antibody that inhibits HER2 signaling pathways) were prepared. [^177^Lu]Lu-DOTA-PEG3k-OPSS was first prepared and then incubated with trastuzumab-PEG_5k_-OPSS and AuNPs to get the final nanoconjugate. [^177^Lu]Lu-AuNPs-PEG-Trastuzumab was more effective than [^177^Lu]Lu-AuNPs (without target functionalization), provoking a decrease in the clonogenic cell survival. As well as an inhibition of the tumor growth was observed after intratumoral injection (3 MBq) in mice bearing HER2-positive tumor xenograft when tumors reached 5–8 mm in diameter (Cai et al. 2017). Moreover, González-Ruíz et al*.* (2017, 2018) developed the ^177^Lu-labeled nanosystem by conjugating AuNPs with the NLS (nuclear localization sequence)—RGD (Arg-Gly-Asp) peptide and an aptamer. The final nanomaterial was prepared to target both α(v)β(3) integrin, and vascular endothelial growth factor (VEGF) overexpressed in the tumor neovasculature. First, the radiolabeled DOTA-GGC peptide is carried out, and then the coupling to AuNPs-NLS-RGD-Aptamer, obtaining the [^177^Lu]Lu-AuNPs-NLS-RGD-Aptamer NPs (29.99 ± 1.90 nm) (González-Ruíz et al. 2017). [^177^Lu]Lu-AuNPs-NLS-RGD-Aptamer decreased cell viability and completely inhibited angiogenesis. Besides, [^177^Lu]Lu-AuNPs-NLS-RGD-Aptamer inhibited tumor progression in mice with glioma tumors (size 0.05 ± 0.01 g) after intratumoral injection (2 MBq) and combined with laser irradiation (Fig. 4) (González-Ruíz et al. 2018). Despite those favorable therapeutic results reported for [^177^Lu]Lu-AuNPs-PEG-Trastuzumab and [^177^Lu]Lu-AuNPs-NLS-RGD-Aptamer, it could be interesting if the authors reproduce the in vivo studies using intravenous injection of the nano-radiopharmaceuticals to be closer to a possible clinical application.

Additionally, Mendoza-Nava et al. (2017) reported a hybrid nanosystem combining AuNPs and DN that also exhibited properties suitable for radionuclide therapy, optical imaging, and plasmonic–photothermal therapy under laser irradiation when the nanosystem is internalized in breast cancer cells. This nanoprobe ([^177^Lu]Lu-DNAuNPs-folate-bombesin) was prepared by conjugating [^177^Lu]Lu-DN to folate and bombesin peptides with AuNPs in the dendritic cavity to target both gastrin-releasing peptide receptors (GRPr) and folate receptors (FR), respectively. Cell viability assays showed that [^177^Lu]Lu-DNAuNPs-folate-bombesin is about four times more lethal than [^177^Lu]Lu-DNAuNPs, without bombesin (targets GRPr) and folate (targets FR) functionalization (Mendoza-Nava et al. 2017). This result evidences the potential effect of targeted radionuclide therapy.

Different types of NPs (AuNCs, and nMOFs) have been radiolabeled using chelates other than DOTA for ^177^Lu complexation with high radiolabeling stability. Pei et al*.* (2021a; b) studied the efficiency of radiolabeled glutathione (GSH) modified AuNCs (~ 2.5 nm) by chelation between ^177^Lu and GSH in the last step of the radiopharmaceutical preparation. To evaluate the therapeutic effect, [^177^Lu]Lu@AuNCs was intratumoral injected (2.8 MBq) when the volume of tumors reached ~ 75mm^3^. It effectively eliminated primary tumors and suppressed distant tumors’ growth when combined with immune checkpoint inhibitors, using the anti-programmed death-ligand 1 (αPD-L1), in mice bearing with breast or colon tumors. The radiolabeled AuNCs showed low physiological toxicity, distributing only in tumors and bladder after intratumoral injection. It was also demonstrated that the combination of radionuclide therapy ([^177^Lu]Lu@AUNCs) and immunotherapy (αPD-L1) significantly suppress the growth of spontaneously metastatic tumors and lengthen the survival time of the transgenic mice (Pei et al. 2021b). On the other hand, Tao et al*.* (2021) studied the radiolabeled PEG modified zirconium-based nMOFs (PCN-224) (~ 140 nm) by chelation between ^177^Lu and porphyrin structure, also in the last step of the radiopharmaceutical preparation. [^177^Lu]Lu-PCN-PEG exhibited high uptake in liver, spleen, kidneys, and tumor at 24 h after intravenous administration in breast tumor-bearing mice. Moreover, [^177^Lu]Lu-PCN-PEG reached high tumor accumulation after intravenous injection (5.55 MBq), resulting in significant inhibition of tumor growth and prolonged survival time without inducing any perceptible toxicity to the treated mice (Tao et al. 2021). Thus, neither [^177^Lu]Lu@AuNCs nor [^177^Lu]Lu-PCN-PEG were functionalized for binding to a specific target in tumors. However, they enhanced radionuclide therapy outcomes highlighting the favorable properties of NPs due to the EPR effect.

Conversely, Cvjetinović et al*.* (2021) demonstrated that ^177^Lu-labeled glucose-modified liposomes (97.3 ± 4.1 nm) exhibited significantly better tumor uptake and prolonged retention than ^177^Lu-labeled non-glucose liposomes (84.9 ± 3.6 nm) after intravenous injection into colon tumor-bearing mice. Hence, the authors concluded that the effect of solely passive EPR on the liposomal accumulation in tumor tissue is relatively low, while the functionalization with glucose enhanced the accumulation by glucose transporters and subsequent endocytosis (Cvjetinović et al. 2021). Therefore, the passive targeting of NPs in cancer by the EPR effect may not be enough in some cases for an efficient tumor accumulation. Thereby, the surface functionalization of NPs with specif-target moieties may overcome the previous limitation.

On the other hand, Chakravarty et al*.* (2020) reported the synthesis and evaluation of intrinsically radiolabeled [^177^Lu]Lu_2_O_3_ NPs entrapped in a protein scaffold ([^177^Lu]Lu_2_O_3_-HSA) through an HSA-mediated biomineralization process. [^177^Lu]Lu_2_O_3_-HSA nanocomposite (4.1 ± 1.2 nm) was rapidly and highly accumulated in melanoma tumors after intravenous injection with significant retention up to 7 days. Also, [^177^Lu]Lu_2_O_3_-HSA nanocomposite greatly retarded tumor growth on a one-time intravenous administration dose (37 MBq) without degenerating liver and kidney. Besides, biochemical and hematological parameters were unaffected, and no behavioral or phenotype changes were observed (Chakravarty et al. 2020).

Finally, Imlimthan et al*.* (2021) recently described a complete study about the theranostic potential of ^177^Lu-labeled CNC loaded with vemurafenib, a clinically approved tyrosine kinase inhibitor, using a murine model of metastatic lung melanoma. For preparing the [^177^Lu]Lu-CNC-V nanoparticles, CNC was radiolabeled by ^177^Lu-DOTA complexation, followed by drug loading in a one-pot reaction. [^177^Lu]Lu-CNC-V (9–14 nm width; 136–158 nm length) showed high retention in the metastatic lung up to 72 h post intravenous injection as well as high uptake in spleen and liver. The survival studies demonstrated its therapeutic potential for treating pulmonary metastatic melanoma through the synergist result of V chemotherapy and ^177^Lu radiotherapy. The therapeutic effects of [^177^Lu]Lu-CNC-V (2 MBq of ^177^Lu and 3.5 mg.kg^−1^ of V) were evaluated after the intravenous administration of the nanosystem, after 14 days of tumor inoculation, followed by a second round of treatment ten days later. Mice treated with [^177^Lu]Lu-CNC-V NPs displayed the longest median survival time of 27 days after treatment, followed by cohorts treated with the [^177^Lu]Lu-CNC (17 days), free V (13 days), and vehicle (12 days) without observing acute toxicity (Imlimthan et al. 2021). Studies like this are fundamental before a clinical translation.

### ^***223***^***Ra-based radiolabeled nanomaterials/micromaterials***

^223^Ra (11.4 d) is a member of the natural Uranium-235 (^235^U) decay chain and was discovered by T. Godlewski as a successive product of Actinium (Ac) decay and identified it as AcX analogically to the previously reported ThX (^224^Ra) (Godlewski 1839, 1905). Artificial preparation of ^223^Ra was performed by neutron irradiation of ^226^Ra (1600 y), leading to ^227^Ra (42 min) that decays to ^227^Ac (21.7 y) a mother nuclide of ^227^Th (18.7 d) and finally the ^223^Ra (Peterson et al. 1949). Thus the ^223^Ra generator based on ^227^Ac can be constructed (Guseva et al. 2004).

$$^{226} {\text{Ra}}\left( {n,\gamma } \right)^{227} {\text{Ra}}\to ^{{\beta^{ - } }} \,^{227} {\text{Ac}}\to ^{{\beta^{ - } }} \,^{227} {\text{Th}}\to ^{\alpha } \,^{223} {\text{Ra}}$$

The EMA and FDA approved the first new-era clinical use of ^223^Ra for the therapy of metastatic castration-resistant prostate cancer (MCRPC). This was the first approved pharmaceutical based on an alpha emitter on the market. However, its use is quite limited due to its self-targeting, mainly to bone tissues mimicking the calcium metabolism (Pharmacopoeia 2014). Attempts to prepare a chelator or other binding moiety for Ra and to label advanced targeting molecules like peptides or antibodies are still challenging the scientific community since the coordination chemistry of radium was not the subject of investigation for decades. Relevant studies appeared just recently employing EDTA (2,2′,2″,2‴-(Ethane-1,2-diyldinitrilo)tetraacetic acid or ethylene diamine tetra acetic acid), macrocyclic ligands, or polyanionic molecules like polyoxopalladates or even liposomes (Henriksen et al. 2002, 2004; Matyskin et al. 2017; Gott et al. 2019; Abou et al. 2021). Completely different approach in targeted alpha therapy that employs preferentially inorganic nanomaterials was proposed to overcome the lack of suitable and stable Ra ligands. A significant step forward was the binding of radium by its encapsulation or surface sorption in the NPs of suitable composition or by the sorption of Ra on the surface of various nanomaterials. An overview of selected ^223^Ra chelator-free labeled nanomaterials is given in Table 5. The inspiration from the naturally occurring alkali-earth element minerals with low solubilities like gypsum (CaSO_4_), celestine (SrSO_4_), or barite (BaSO_4_) (Rosenberg et al. 2018) could be expected. Ionic size and chemistry of Ra allow various methods for its incorporation like isostructural incorporation, pores intrusion, ion exchange, etc.

**Table 5:** Selected potential nano/micro-materials labeled with ^223^Ra

^223^Ra labeled NPs	Particle size	Labeling method	Stage of research	References
Hydroxyapatite	21.7 ± 6.9 nm (TEM)	Surface sorption, Intrinsic labeling	In vitro*,* radiochemical analysis	Kukleva et al. 2019; Suchánková et al. 2020b)
CaCO_3_	3–30 μm (light scattering)	Surface sorption	In vivo*,* rodents	Li et al. 2020)
Fe_3_O_4_	4–26 nm (TEM) 284 nm (DLS)	Surface sorption	In vitro*,* radiochemical analysis	Mokhodoeva et al. 2016)
Barium ferrite	14–30 nm (TEM)	Intrinsic labeling	In vitro*,* cell lines	Gawęda et al. 2020)
LaPO_4_	3–10 nm (TEM)	Surface sorption, Intrinsic labeling	In vitro*,* radiochemical analysis	Toro-González et al. 2020)
TiO_2_	5.3 ± 1.7 nm (TEM)	Surface sorption, Intrinsic labeling	In vitro, radiochemical analysis	Kukleva et al. 2019; Suchánková et al. 2020b)
BaSO_4_	140 ± 50 nm (TEM/DLS)	Intrinsic labeling	In vitro, radiochemical analysis	Reissig et al. 2019)
GdVO_4_	length: 23–48 nm, width: 16–32 nm (TEM, pH dependent)	Intrinsic labeling	In vitro*,* radiochemical analysis	Toro-González et al. 2020)
Nanozeolite	30–800 nm (SEM) 226.1 ± 44.2 nm (DLS)	Intrinsic labeling	In vivo, rodents	Czerwińska et al. 2020; Lankoff et al. 2021)
Nanodiamonds Graphene oxide Nanotubes	3–10 nm > 100 nm (HR-TEM) 30 nm	Surface sorption	In vitro radiochemical analysis	Kazakov et al. 2020))
Nanomicelles	129.4 nm ± 0.3 (DLS)	Encapsulation	In vitro,* cell lines*	Yang et al. 2022)

It is important to mention that the intended use of nanomaterials also had second motivation. That was to solve the problem of the daughter radioactive nuclei release from the targeting molecules due to the nuclear recoil effect since their spread over the body causes unwanted irradiation of healthy tissues. In this way, at least partial retention of radioactive progeny should improve the overall therapeutic outcome (Kozempel et al. 2018). On the other hand, controlled release of daughter progeny from a point source in close vicinity of tumors was reported to improve the treatment outcome in so-called DART (diffusing alpha-emitters radiation therapy) localized tumor therapy (Popovtzer et al. 2020; Keisari and Kelson 2021) and could be possibly transferred to other alpha particle therapy modalities (Perrin et al. 2022).

In addition to the properties of the neat nanomaterials used for successful Ra binding, the surface of the nanomaterials offers a possibility of additional modification such as protective coating, binding of active targeting moieties, attaching chelators, etc. (Trujillo-Nolasco et al. 2021). Even though the results of in vitro tests of labeled nanomaterials may indicate very promising findings, their translation into advanced in vivo preclinical and clinical stages of research is not straightforward. It would definitely bring novel obstacles and challenges for their systemic application, e.g., an unspecific uptake in RES, problematic active targeting, barriers crossing, toxicity, etc. (Lankoff et al. 2021; Kleynhans et al. 2021). However, further research is needed to elucidate the overall fate of the radiolabeled nanomaterials in vivo*,* such as active or passive transport to the tumor, the tumor microenvironment modification, immunogenic tumor-cell death, etc.

Unfortunately, there are still only very few in vivo studies available on the Ra-labeled nanomaterials intended for use in nuclear medicine. This may relate to the previous, relatively low availability of Ra for research purposes. Translation of nanomaterials to clinical trials/practice is thus the next important step in future research. This research could be promoted by the Good Manufacturing Practices grade ^223^Ra readily available on the market. It could be expected that due to future implementation of other MCRPC treatment protocols employing ^177^Lu, ^225^Ac and ^227^Th labeled PSMA derivatives or antibodies (Kratochwil et al. 2016; Rosar et al. 2021; Hagemann et al. 2020; Juzeniene et al. 2021) together with restricted Ra chloride palliation, therapy (EMA/500948/2018 2018), its availability may further increase for advanced therapies research based on Ra. Promising results in the study of malignant ovarian epithelial tumors have been reported by using another alpha-emitting radium isotope—^224^Ra (3.66 d). For this purpose, ^224^Ra-labeled calcium carbonate microparticles were prepared (Westrøm et al. 2018). Studies on ES-2 and SKOV3-luc models were performed, and intraperitoneal treatment with ^224^Ra-microparticles gave a significant antitumor effect with either considerably reduced tumor volume or a survival benefit. The combination of ^223^Ra (or ^224^Ra) and nanomaterials or micromaterials yields multimodality, which may bring an interesting therapeutical effect with a safety profile at an effective dose. This alpha radiation tool seems to be still promising for loco-regional treatment.

## Liver radioembolization as a successful experience using radiolabeled microspheres

Radioembolization with radiolabeled microspheres is a radiation-based therapy modality used to treat primary liver tumors and metastases, which are untreatable by surgery or chemotherapy. The treatment consists in the employment of microspheres that contain therapeutic radioisotopes (β-emitters such as yttrium-90 or holmium-166) (Spa et al. 2018). Although several clinical trials have demonstrated the efficacy of radioembolization (Hilgard et al. 2010; Kennedy et al. 2012; Rosenbaum et al. 2013), the displacement of a fraction of the administered particles towards the microvasculature of the lung instead of the liver remains a challenge. In order to overcome this issue, several approaches and new nanosystems have been proposed. For instance, Zhao et al. (2016b) have proposed the use of chelate-free radioactive nanoparticles taking advantage of radioisotopes and their non-radioactive isotopes of the same element as integral components of nanoparticles. In this direction, they synthesized chelate-free ^64^Cu-doped copper sulfide nanoparticles with a mean size of 11.7 nm and with high radiochemical yield. Also, Jamre et al. (2018) have prepared carrier-free ^188^Re loaded poly (L-lactic acid) (PLLA) microspheres through ^188^Re sulfide colloidal nanoparticles (^188^Re -SCNPs). The microspheres presented a modal size of 29 μm and radiolabeling efficiency > 99%. The biodistribution after intravenous injection in healthy BALB/c mice showed high accumulation in lung as a first capture pathway organ for microsphere.

## Toxicity of nanoparticles

The toxicity of nanoparticles is a concern and may limit its use. Several factors have an influence on the toxicity of nanoparticles, like size, shape, surface, charge composition, solubility, and aggregation. Due to their high surface area, nanoparticles can easily interact with cellular components such as nucleic acids, proteins, fatty acids, and carbohydrates. Also, the small size facilitates cell entrance, which may result in nucleus interaction as the influence in several inner mechanisms/organelles of the cell, for instance, the mitochondria. Also, the surface charge of nanoparticles has a pronounced effect. The higher the nanoparticle’s positive charge, the greater electrostatic interactions it has with the cell and, thus, greater endocytic uptake (Sengul and Asmatulu 2020; Niazi et al. 2009; Huang et al. 2017).

Many in vitro and in vivo studies have shown that exposure to nanoparticles could induce the production of reactive oxygen species (ROS). ROS generation is directly related to alteration in mitochondrial metabolism, which represents one of the main markers confirming apoptosis induction since ROS causes oxidative stress, inflammation, and subsequent damage to proteins, cell membranes, and DNA (Huang et al. 2017; Freire et al. 2021; Wigner et al. 2021).

Helal-Neto et al. (2020) evaluated the toxicity effect of polylactic acid (PLA) nanoparticles and magnetic core mesoporous silica nanoparticles (MMSN) of 1000 nm and 50 nm, respectively. The nanoparticles were analyzed in the following cell lines: melanoma (MV3), breast cancer (MCF-7, MDA-MB-213), glioma (U373MG), prostate (PC3), gastric (AGS) and colon adenocarcinoma (HT-29), melanocyte (NGM), fibroblast (FGH) and endothelial (HUVEC), evaluating cell migration, tubulogenesis, tubulin, AKT, GADPH, ERK, actin skeleton, and several other parameters. The results demonstrated that neither PLA nor MMSM nanoparticles could produce a toxic effect. Controversially, Wigner et al. (2021) evaluating the influence of polymeric nanoparticles (PLA/MMT/TRA, PLA/EDTMP, PLGA/MDP, and Pluronic F127 Ms) on the cell, homeostasis demonstrated that all nanosystems were able to produce a toxic effect, which included: genotoxicity effect by internucleosomal DNA fragmentation and formation of ROS. In the same way, Freire et al. (2021), studying the biomedical application of graphitic carbon nitrides nanoparticles, found that although graphitic carbon nitrides may induce cell apoptosis, the mechanism was not by the formation of ROS formation.

Regarding the toxicity of metallic nanomaterial, it depends on the oxidation state, ligands, solubility, and morphology as the health conditions of the subject. Although the complete mechanism where metallic nanoparticles produce toxic events is unknown, researchers believe that metallic nanoparticles can be toxic due to the release of ions and disbursing throughout the body (Długosz et al. 2020). The number of ions released generates a cascade of events, culminating with a high amount of ROS, leading to increased inflammation, mitochondrial perturbation secretion of lactate dehydrogenase, damage to DNA, proteins, and lipids ended in death by apoptosis or necrosis (Rasmussen et al. 2010). A study by Yao et al. (2019) has shown that metal nanoparticles and metal oxides nanoparticles (nano-Cu, nano-Ag, nano-Ni, nano-TiO2, nano- ZnO, and nano-Au) have a high accumulation in the liver and the mononuclear phagocytic system after reaching the systemic circulation, which resulted in the interaction of these nanoparticles with hepatic cells, with the possibility of changing the structure and function of hepatocytes, Kupffer cells, liver sinusoidal endothelial cells, hepatic stellate cells, and others. This is corroborated by Attarilar et al. (2020) that have discussed in a review study that the main mechanism involved in toxicity of metallic nanoparticles are: i) ROS formation, ii) cell damage by membrane perforation, iii) cytoskeleton damage, iv) mutagenesis, v) mitochondrial damage and vi) lysosome damage. It is important to notice that there is no information regarding specific toxicity of radioactive metallic nanoparticles, and it could be an important field of study.

The functionalized metal NPs can be either actively or passively delivered to the target site for specific therapy. Thus, the fabrication and functionalization of nanomaterials can be effectively carried out for attaining antimicrobial and anticancer properties. Functionalization modifies the physicochemical properties of nanomaterials thereby altering toxicity to a minimal level, enhancing protein adsorption, and affecting cellular activity. Also, functionalization increases the solubility of nanomaterials and their escape from primary immune reactions that results in strengthening the possibility of using nanomaterials as carriers of biological and therapeutic molecules without affecting the immune system (Veerapandian et al. 2014).

## Discussion

Nanoparticles used for biomedical applications have several advantages compared to conventional drugs. It is worth highlighting the improvement of bioavailability, the increased biological half-life, increased targeting, and higher bioaccumulation. Nanoparticles show a surface-to-volume ratio, which allows the encapsulation of diverse therapeutics molecules: radionuclides, contrast agents, aptamers, peptides, and many other compounds (Corrêa et al. 2022; Magne et al. 2021b; Jeong et al. 2018; Kim et al. 2017). Due to the high surface area, physical adsorption or electrostatic interactions insert some active ingredients, like radionuclides. Besides that, the high surface allows immobilization of therapeutics by chemistry functionalization, changing the in vivo behavior of this nanoparticle as well as increasing the target (Liu et al. 2020; Yetisgin et al. 2020; Castillo et al. 2018; Welch et al. 2009; Wu et al. 2020b).

A disadvantage of nanoparticles is thatafter reaching the bloodstream, they are prone to aggregation and protein opsonization. Both processes alert the immune systems, leading to a massive clearing of the nanoparticles from the bloodstream with high uptake by the liver, spleen, and kidney. This rapid and non-specific clearance by the immune system results in decreased retention time and thus limits bioavailability (Santos et al. 2017).

There are several advantages and many limitations on the use of nanoparticles. For instance, variations on the surface charge (zeta potetntial), morphology and size may change drastically the behavior of the nanosystems in the cellular and molecular level. Most nanoparticles enter the cells by endocytosis through clathrin- or caveolae-dependent mechanisms (Behzadi et al. 2017). In both cases, the shape of nanoparticles plays an important role in biodistribution and, subsequentially, the internalization by cells. For instance, rod-shaped cationic nanoparticles are easier targets for endosomal uptake than cationic nanoparticles of other shapes, suggesting that these nanoparticles may be comprehended by immune system cells as rod-shaped bacteria (Gratton et al. 2008). Finally, surface charge also plays an essential whole in the biodistribution and targeting of nanoparticles. Positively charged nanoparticles are taken up to more extent by liver hepatocytes when compared to uncharged. Meanwhile, negatively charged nanoparticles show a broader liver distribution (Elci et al. 2016). According to He et al. (He et al. 2010), negative charged NPs tended to accumulate in tumors more efficiently, and Frolich (Fröhlich 2012) stated that positively charged nanoparticles are more cytotoxic than negative variants of similar size.

Therefore, the design of the nanoparticles depends on further application. This review paper revisited the current status of the radiolabeled nanoparticles for molecular imaging and radionuclide therapy. We overview the nanoparticles labeled with imaging (^99m^Tc and ^64^Cu) and therapeutic (^177^Lu and ^223^Ra) radiometals. Unfortunately, most of these radiolabeled NPs have only been assessed at preclinical settings, while just a few are clinically approved. The ^99m^Tc-labeled NPs for sentinel lymph node, the ^99m^Tc-labeled microparticles for lung perfusion imaging as well as the ^90^Y/^166^Ho-labeled microspheres for liver radioembolization were the first clinically approved a few years ago, and the unique that is in the clinic to date, to the best of our knowledge.

^64^Cu-NPs have some challenges: they must have superior kinetic inertness to Cu(II) decomplexation (proton-assisted as well as transchelation or transmetallation) to avoid undesirable uptake in healthy tissues (e.g., liver) when is injected into a living organism (Wadas et al. 2007). Hence, the stable Cu complexation is sometimes a crucial challenge in the ^64^Cu radiopharmaceuticals. Nevertheless, to the best of our knowledge, we did not find any findings regarding this in the case of the ^64^Cu-labeled NPs. Perhaps because many NPs can be eliminated with the physiological uptake in healthy tissue (liver, spleen) due to the opsonization, or maybe because of the high in vivo stability of ^64^Cu-labeled NPs. Still, ^64^Cu-labeled NPs displayed promising outcomes at preclinical settings for monitoring the efficacy of therapies, like chemotherapy, and for new treatment planning using molecular imaging. Currently, there is a clinical trial phase 1 under recruiting (NCT04167969) to evaluate ^64^Cu-labeled NPs to guide the surgical treatment of prostate cancer (NCT04167969).

**Fig. 5:**
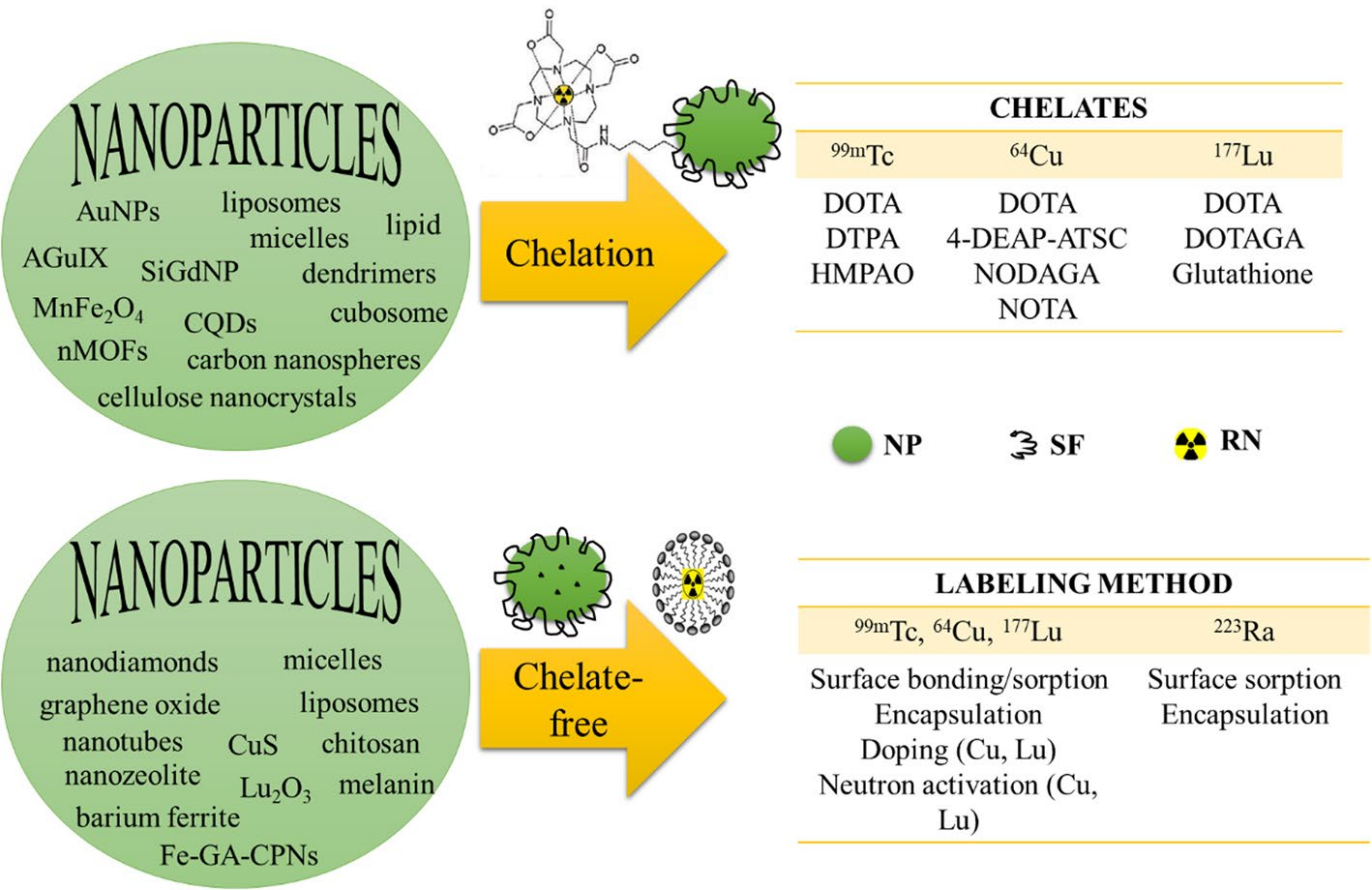
Radiolabeling of nanoparticles using chelate or chelate-free approaches. NP, nanoparticle; SF, surface functionalization; RN, radionuclide (^99m^Tc, ^64^Cu, ^177^Lu, ^223^Ra); AuNPs, gold nanoparticles; AGuIX, gadolinium nanoparticles; SiGdNP, silica gadolinium nanoparticles; MnFe_2_O_4_, superparamagnetic manganese ferrite; CQDs, carbon quantum dots; nMOFs, nanoscale metal–organic frameworks; Fe-Ga-CPNs, iron-gallic acid coordination nanoparticles

Unlike ^99m^Tc, ^64^Cu, and ^177^Lu, the stable chelation of the alkaline earth metal ^223^Ra is a challenge (Abou et al. 2021; Lankoff et al. 2021) because of its complete electronic configuration ([Rn]7s^2^) and the recoil energy effect. Hence, properly designed encapsulating of ^223^Ra in nanomaterials such as micelles (Hilgard et al. 2010) or surface sorption onto NPs might be the solution to get new ^223^Ra radiopharmaceuticals for alpha-targeted therapy. Also, these strategies might solve the problem of the daughter radioactive nuclei release from the ^223^Ra-labeled molecules. Although a few findings with ^223^Ra-labeled NPs (very little) have been reported, still in vivo evidence that validates the previous hypotheses is a lack. Figure 5 shows the main approaches used for radiolabeling the overviewed NPs using or not chelate-complexation, both with high radiochemical stability (reported in most studies).

As previously mentioned, nanoparticles have excellent properties for designing therapeutic radiopharmaceuticals: high surface-to-volume ratio, easy surface modification, EPR effect, improved bioavailability, and increased biological half-life. For in vivo applications, toxicity might be the most increased limitation of NPs. However, PEGylation might overcome that limitation. Among the reviewed works, ^177^Lu-labeled NPs are the most preclinically evaluated for radionuclide therapy and theranostics with positive therapeutic effects and low perceptible physiological toxicity. Target-specific functionalization enhanced tumor accumulation and retention as well as the therapeutic effect. Moreover, some ^177^Lu-labeled NPs combined radionuclide therapy with other therapies such as chemotherapy and immunotherapy in one-pot delivery. Still, some preclinical studies used intratumoral injection instead of intravenous to evaluate the therapeutic effect of the ^177^Lu-labeled NPs. As a proof-a-concept, the intratumoral injection may be accepted. However, in vivo studies using intravenous injection are needed to evaluate the biodistribution and pharmacokinetics as well as to demonstrate better the safe and effective use of the radiolabeled NPs for cancer therapy. In addition, we suggest the use of metastatic preclinical models to evaluate their therapeutic effect and safety in a closer approximation to the clinical settings before clinical translation. To the best of our knowledge, we did not find ongoing clinical trials with ^177^Lu-labeled NPs yet.

Therefore, the lack of clinical outcomes, mainly in the last five years, limits us to conclude that the radiolabeled nanomaterials for biomedical applications are the future of radiopharmacy. Despite the advantages of the nanoparticles over macromolecules, there is a long way to go and much more work to do for demonstrating the future use of the radiolabeled nanoparticles in radiopharmacy.

## Conclusions and outlook

In this review, the data demonstrated that in some cases, the use of radiolabeled nanoparticles might increase the quality of the therapy as well as the imaging. The development of theranostic nanoparticles may represent an important advance in the radiopharmacy field and may represent the last frontier. Although several benefits have been described in the use of radioactive nanoparticles, there are also several limitations. One of the most prominent limitation is the rapid recognition of nanoparticles (radioactive or not) by the mononuclear phagocyte system, leading to the rapid elimination of nanoparticles from the bloodstream. Another issue is the corona protein formation, which also leads to accelerated elimination and inactivation of the nanoparticles. Finally, the toxicity of metals and radioactive metals must be underlined since several particularities must be better understood.

In this direction, some outlooks are proposed:

Understand the stability of organic and inorganic nanoparticles, especially with beta and alpha emitters radionuclides;

Understand the differential toxicity of metals and radioactive metals;

Think in new forms to avoid the mononuclear phagocyte system. Promising results were recently reported with NPs using the differential esterase activity in organs (Lee et al. 2021) or enzyme-powered nanomotors (Hortelao et al. 2021).

## Data Availability

Data sharing is not applicable to this article as no datasets were generated or analysed during the current study.

## Abbreviations

^125^I: Iodine-125
^131^Ba: Barium-131
^14^C: Carbon-14
^177^Lu: Lutetium-177
^199^Au: Gold-199
^223^Ra: Radium-223
4-DEAP-ATSC: Diacetyl 4,4′-bis(3-(N,N-diethylamino)propyl)thiosemicarbazone
^64^Cu: Copper-64
^68^ Ga: Gallium-68
^89^Zr: Zirconium-89
^90^Y: Yttrium-90
^98^Mo: Molybdenum-98
^99m^Tc: Technetium-99m
AGuIX: Gadolinium nanoparticles
ATSM: Diacetyl bis(N 4-methylthiosemicarbazone)
AuNCs: Gold nanoclusters
AuNPs: Gold nanoparticles
Barite: BaSO_4_
CB-TE2A: 4,11-Bis(carboxymethyl)-1,4,8,11-tetraazabicyclo[6.6.2]hexadecane
Celestine: SrSO_4_
CH: Chitosan
CNC: Cellulose nanocrystals
CNS: Carbon nanospheres
CQDs: Carbon quantum dots
CT: Computed tomography
Cubosomes: Lipidic cubic-phase nanoparticles
CuS: Copper sulfide
cys-DB: Cysteine-diabody
DART: Diffusing alpha-emitters radiation therapy
DN: Dendrimers
DOTA: 1,4,7,10-Tetraazacyclododecane-1,4,7,10-tetraacetic acid
DOX: Doxorubicin
DTPA: Diethylenetriamine pentaacetate
EDTA: 2,2′,2″,2‴-(Ethane-1,2-diyldinitrilo)tetraacetic acid or ethylene diamine tetra acetic acid
EMA: European Medicines Agency
EPR: Enhanced permeability and retention
FDA: Food and Drug Administration
Fe_3_O_4_–SiO_2_: Iron oxide bound to silica
Fe-GA-CPNs: Iron-gallic acid coordination nanoparticles
FR: Folate receptors
GRPr: Gastrin-releasing peptide receptors
GSH: Glutathione
Gypsum: CaSO_4_
HER2: Human epidermal growth factor receptor 2
HMPAO: D,l-Hexamethylene-propyleneamine oxime
HSA: Human serum albumin
i.v.: İNtravenous
IUPAC: International Union of Pure and-Applied Chemistry
LET: Linear energy transfer
LND: Lipid nanodiscs
LNPs: Lipid nanoparticles
Lu_2_O_3_: Rare sesquioxides
MAA: Macroaggregated albumin
Macrin: Polyglucose nanoparticles
MCRPC: Metastatic castration-resistant prostate cancer
MMSN: Magnetic core mesoporous silica nanoparticles
MnFe_2_O_4_: Superparamagnetic manganese ferrite
MRI: Magnetic resonance imaging
MSNPs: Mesoporous silica nanoparticles
NLS: Nuclear localization sequence
nMOFs: Nanoscale metal–organic frameworks
NODAGA: 1,4,7-Triazacyclononane, 1-glutaric acid-4,7-diacetic acid
NOTA: 1,4,7-Triazacyclononane-N,N′,N″-trisacetic acid
NPs: Nanoparticles
PCN-224: Zirconium-based nMOFs
PD-1: Programmed cell death-1
PEG: Polyethylene glycol
PET: Positron emission tomography
PLA: Polylactic acid
PLLA: Poly(l-lactic acid)
PTX: Paclitaxel
RES: Reticuloendothelial system
RGD: Arg-Gly-Asp
ROS: Reactive oxygen species
SCNPs: Sulfide colloidal nanoparticles
SiGdNPs: Silica gadolinium nanoparticles
SLNs: Solid lipid nanoparticles
SPECT: Single-photon emission computed tomography
SPION: Superparamagnetic iron oxide
TETA: 1,4,8,11-Tetraazacyclotetradecane-1,8-diacetic acid
UPS: Ultra-pH sensitive
V: Vemurafenib
VEGF: Vascular endothelial growth factor
